# C2L3-Fusion: An Integrated 3D Object Detection Method for Autonomous Vehicles

**DOI:** 10.3390/s25092688

**Published:** 2025-04-24

**Authors:** Thanh Binh Ngo, Long Ngo, Anh Vu Phi, Trung Thị Hoa Trang Nguyen, Andy Nguyen, Jason Brown, Asanka Perera

**Affiliations:** 1 Department of Electrical and Electronic Engineering, University of Transport and Communications, Hanoi 100000, Vietnam; trangntth@utc.edu.vn; 2 Software and Service Development Department, Mobifone Digital Services, Hanoi 100000, Vietnam; long.ngo@mobifone.vn; 3 Computer Science Department, College of Engineering, Michigan State University, East Lansing, MI 48823, USA; phivu@msu.edu; 4 School of Engineering, University of Southern Queensland, Springfield, QLD 4300, Australia; andy.nguyen@unisq.edu.au (A.N.); jason.brown2@unisq.edu.au (J.B.)

**Keywords:** AI, deep learning, C2L3-Fusion, 2D detection, 3D detection, autonomous vehicle

## Abstract

Accurate 3D object detection is crucial for autonomous vehicles (AVs) to navigate safely in complex environments. This paper introduces a novel fusion framework that integrates Camera image-based **2D object detection using YOLOv8** and LiDAR data-based **3D object detection using PointPillars, hence named C2L3-Fusion**. Unlike conventional fusion approaches, which often struggle with feature misalignment, **C2L3-Fusion** enhances spatial consistency and multi-level feature aggregation, significantly improving detection accuracy. Our method outperforms state-of-the-art approaches such as YoPi-CLOCs Fusion Network, standalone YOLOv8, and standalone PointPillars, achieving mean Average Precision (mAP) scores of **89.91% (easy), 79.26% (moderate), and 78.01% (hard)** on the KITTI dataset. Successfully implemented on the Nvidia Jetson AGX Xavier embedded platform, **C2L3-Fusion** maintains real-time performance while enhancing robustness, making it highly suitable for self-driving vehicles. This paper details the methodology, mathematical formulations, algorithmic advancements, and real-world testing of C2L3-Fusion, offering a comprehensive solution for 3D object detection in autonomous navigation.

## 1. Introduction

In the modern era, automation has emerged as a cornerstone for technological advancement, transforming industries from manufacturing to transportation. Among these, autonomous vehicles (AVs) stand out as a revolutionary development, offering significant improvements in transportation safety and efficiency. The potential to reduce traffic accidents, mitigate congestion, and lower environmental impact has made AVs a focal point of research worldwide [1,2]. Object detection—a critical component of AV systems—enables vehicles to perceive and navigate their surroundings intelligently [3].

Despite significant advances, current object detection methods still face several challenges. Traditional 2D detection techniques using camera systems struggle in low light, glare, darkness, or fog, and lack depth information, which limits accurate distance estimation [4,5]. In these adverse lighting conditions for object detection using cameras, the use of LiDAR for autonomous vehicles is a viable solution [6]. While 2D object detection solutions using low-cost hardware are suitable for object-tracking in autonomous vehicles [7], there is a growing focus on 3D detection solutions to enhance self-driving capabilities. However, camera-based 3D detection also suffers in adverse conditions. LiDAR-based 3D detection offers accurate spatial data but faces issues with sparseness and high computational demands [8]. Although integrating these technologies shows promise, it complicates data fusion, alignment, and real-time processing, which remain active research areas [9,10].

This study aims to address these challenges by developing a novel fusion-based object detection system that combines the strengths of 2D and 3D detection methods. Specifically, we propose a framework that integrates high-resolution camera images with point cloud data from LiDAR to enhance the accuracy and robustness of object detection in autonomous vehicles. Our model is designed for real-time operation on embedded platforms, ensuring practical applicability in AV systems [11,12]. This model has the following notable features:

- Combining 2D image features and 3D LiDAR point clouds to improve precision detection and depth estimation [8,13].
- Algorithmic advancements: Introduction of a new fusion architecture leveraging the CLOCs (Camera-LiDAR Object Candidates) framework for optimal feature integration and robust object detection [14].
- Implementation on embedded systems: Real-time deployment of the model on the Nvidia Jetson AGX Xavier, demonstrating its applicability [12].
- Comprehensive evaluation: Validation of the model on the KITTI dataset, achieving significant improvements in mean Average Precision (mAP) across easy, moderate, and hard scenarios [11].

The remainder of this paper is organized as follows: Section 2 reviews related work on object detection methods in autonomous vehicles. Section 3 presents the proposed methodology, detailing the fusion network architecture and training process. Section 4 discusses the implementation and evaluation results, including real-world applications. Finally, Section 5 concludes with insights and future directions.

## 2. Related Work

The field of 3D object detection for autonomous vehicles has been extensively researched, with existing methods largely categorized into three main groups: image-based detection, point-cloud-based detection, and multi-sensor fusion-based detection. Each of these approaches has its advantages and limitations, which are discussed below, along with the necessity of further research to address the gaps.

### 2.1. Motivation for Combining 2D and 3D Data in Object Detection

The motivation to combine 2D and 3D data in object detection arises from the complementary strengths and limitations of the two modalities. By integrating these data sources, it becomes possible to address key challenges in autonomous vehicle perception systems while achieving a more robust and accurate object detection framework.

**Figure 1:**
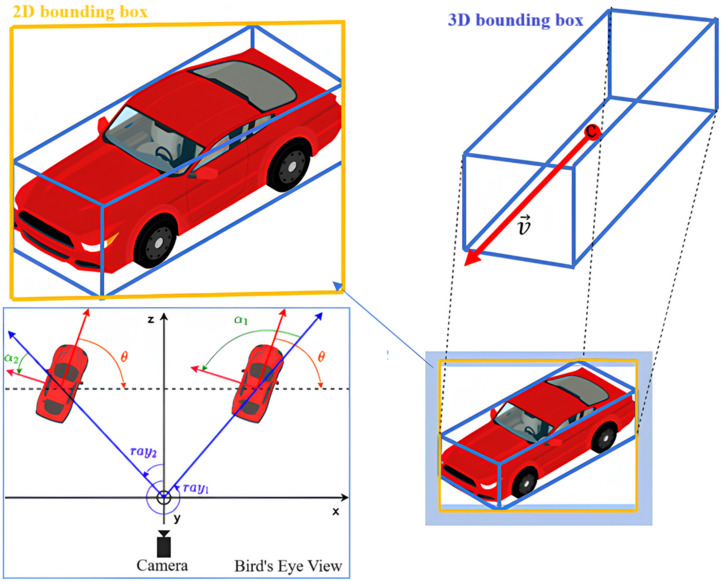
Illustration of merging 2D and 3D bounding boxes with direction vectors to enhance object detection performance.

- Image-based methods rely on RGB cameras, which are effective in capturing detailed semantic information, including texture, color, and shape. However, these methods lack intrinsic depth perception, making it difficult to estimate object distances accurately, especially in occluded, poorly lit, or highly dynamic environments.
- LiDAR sensors provide precise depth and spatial data, essential for accurate 3D localization. Nevertheless, LiDAR data is often sparse, especially for small or distant objects, and lacks the rich semantic information necessary to distinguish between objects of similar shapes. Additionally, processing dense point clouds can be computationally intensive, posing challenges for real-time performance.
- The fusion of 2D and 3D data addresses these limitations by leveraging the advantages of both modalities (see Figure 1):
  – Semantic Enrichment from Images: High-resolution RGB images provide detailed object context, improving class recognition and distinguishing between visually similar objects.
  – Depth and Spatial Precision from LiDAR: LiDAR point clouds deliver accurate 3D localization, enabling robust distance and orientation estimation.
  – Improved Robustness in Complex Scenarios: The combined approach enhances detection performance in challenging environments, such as those with occlusions, varying light conditions, or high object density.

Figure 1 illustrates the integration of information from two types of object detection methods: 2D bounding boxes and 3D bounding boxes. Combining these two data types provides several key advantages for autonomous vehicles, including the following:

- Accurate real-time object localization, by leveraging both the 2D image plane and the 3D spatial geometry of objects.
- Prediction of object motion and behavior, such as determining movement direction (e.g., turning left or going straight), using orientation angles (α), velocity vectors (→v), and the object’s position in space.
- Effective operation in diverse and dynamic environments.

The figure also includes a Bird’s Eye View (BEV) representation, which offers a top-down perspective of the scene from above the vehicle. BEV is typically generated using LiDAR data, which provides dense 3D point clouds that can be projected onto the ground plane. This perspective helps in understanding the relative position, orientation (θ), and trajectory of surrounding objects in a global coordinate system (x, y, z), providing crucial contextual information for path planning and collision avoidance in autonomous driving.

The 2D bounding boxes help detect objects in a 2D image—such as a typical camera frame—by enclosing them in rectangles. In contrast, 3D bounding boxes with direction vectors capture objects in 3D space, specifying not only their size and position but also their orientation and movement direction.

Combining these two modalities significantly enhances object detection performance, particularly in complex scenarios where objects may be partially occluded or closely packed, making detection challenging. By fusing both 2D and 3D information, this approach overcomes the limitations of standalone systems and improves detection accuracy, robustness, and real-time capability.

In essence, the fusion of 2D and 3D bounding boxes with directional cues is a powerful technique to make object detection more reliable and effective in real-world, challenging environments—an essential capability for autonomous vehicle systems.

### 2.2. Image-Based Detection

Image-based methods rely on RGB cameras to predict 2D bounding boxes, which are then extended into 3D using depth estimation. While these methods excel in capturing semantic and texture-rich information, they often face challenges related to accurate depth estimation, particularly in occluded or poorly lit environments.

- Mono3D [4]: This approach uses monocular images to generate 3D bounding boxes by integrating semantics, context, and geometric priors. While Mono3D improves detection precision through Fast R-CNN-based refinements, its reliance on hand-crafted features limits scalability in complex scenarios.
- DSGN [15]: Utilizing stereo image pairs, this method estimates depth and object location independently of specific classes. Despite its effectiveness, real-time implementation is hindered by synchronization issues between stereo cameras.
- MonoDTR [16]: This more recent approach integrates depth-aware transformers, capturing spatial relationships effectively and achieving competitive results on the KITTI dataset. However, its performance is constrained in dynamic, crowded environments due to reliance on monocular depth estimation.

While these methods provide rich semantic context, they are fundamentally constrained by depth estimation inaccuracies, which our fusion-based approach aims to resolve.

### 2.3. Point-Cloud-Based Detection

Point-cloud-based methods use LiDAR data to create spatially accurate 3D object representations. These methods are critical for tasks requiring precise localization and depth information but often face challenges related to data sparsity and computational overhead.

- PointPillars [17]: This method converts LiDAR pointing clouds into pseudo-images for processing with 2D CNNs, achieving a balance between efficiency and accuracy. However, the voxelization process can introduce information loss.
- PV-RCNN [18]: This hybrid model combines voxel-based and point-based features for improved detection performance, excelling in dense urban environments.
- Voxel R-CNN [19]: This voxel-based architecture refines proposals through a lightweight design, achieving competitive performance on the nuScenes dataset while maintaining computational efficiency.

Although these methods provide reliable depth estimation, they require optimizations to effectively handle sparse data, especially for small or distant objects. Our proposed approach enhances detection performance by integrating LiDAR-based features with image-based contextual information.

### 2.4. Multi-Sensor Fusion-Based Detection

- Multi-View Fusion Methods: As the information of a single view (e.g., the front view image or BEV point cloud) is usually not sufficient for understanding real scenes, some researchers try to explore multi-view fusion to improve the performance of 3D object detection tasks. AVOD [20] refine the detection box by fusing BEV and camera feature maps for each ROI region. Although these multi-view approaches usually outperform single-view-based methods, they still suffer from information loss due to the process of converting a point cloud to a specific view.
- Voxel and Image Fusion Methods: Many recent LiDAR-only methods convert the raw LiDAR point cloud to regular voxel grids for 3D object detection, thanks to its effectiveness and efficiency. To further improve the robustness of 3D detectors, some researchers [21,22,23,24] devote their efforts to voxel-based and camera image fusion methods. Specifically, ConFuse [25] proposes a novel continuous fusion layer, which not only achieves the voxel-wise alignment between BEV and image feature maps but also captures local information to improve the detection performance. MVX-Net [21] proposes to enhance the voxel feature representations with semantic image features by fusing the features of the camera image and LiDAR point cloud in the early stage. By utilizing a cross-view spatial feature fusion strategy, 3D-CVF [22] effectively fuses the spatial feature from both the camera image and LiDAR point cloud. Due to the quantized error brought by the voxelization operation, these methods have limitations in establishing the accurate corresponding relationship between the camera image and LiDAR point cloud.
- Raw Point Cloud and Image Fusion Methods: Considering that the point cloud possesses rich geometric structure information but lacks plentiful semantic information, some researchers [25,26,27] have tried to fuse the raw point cloud and the camera image. Specifically, PointFusion [27] and SIFRNet [28] first extract semantic features and produce 2D proposals from camera images using off-the-shelf 2D detectors [29,30,31]. The extracted semantic features are then combined with the point features extracted from the corresponding frustum to generate 3D bounding boxes. PointPainting [25] enriches each point feature with the corresponding output class scores predicted by a pre-trained image semantic segmentation network. PI-RCNN [32] employs a segmentation sub-network to extract full-resolution semantic feature maps from images and then fuses the multi-sensor features via a powerful PACF attention module. ImVoteNet [33] further improves the detection performance of VoteNet [34] by lifting 2D camera image votes as well as geometric, semantic, and texture cues from an off-the-shelf 2D detector and then combining them with 3D votes in point clouds.

While these methods improve detection accuracy, they often fail to meet the real-time requirements of autonomous systems. The C2L3-Fusion proposed in this study is a novel fusion framework designed for both high accuracy and real-time performance. Our approach integrates two state-of-the-art models:

- YOLOv8 for 2D object detection using RGB cameras. YOLOv8 is known for its exceptional speed and accuracy, making it one of the best choices for real-time vision tasks.
- PointPillars for 3D object detection using LiDAR. This model efficiently processes point clouds to generate precise 3D bounding boxes with minimal computational overhead.

## 3. Methodology

### 3.1. System Design and Fusion Network Architecture

This section describes the methodology used in our research, detailing the sensor fusion framework, data processing pipeline, and evaluation methods.

(1) Sensor Fusion Framework

To enhance perception accuracy, we integrate multiple sensors, specifically LiDAR and cameras, using a fusion approach. The fusion process consists of the following steps:

- Preprocessing: Each sensor’s raw data is independently processed to remove noise and extract key features.
- Time Synchronization: A synchronization mechanism ensures that data from different sensors correspond to the same timestamps.
- Fusion Strategy: Integration is performed using a deep-learning-based method that combines YOLOv8 for 2D object detection and PointPillars for 3D object detection.

(2) Data Processing Pipeline

- 2D Object Detection (YOLOv8):
  – Backbone: Extracts low- and high-level features from RGB images.
  – Neck: Combines multi-scale features to enhance detection robustness.
  – Head: Produces 2D bounding boxes, class probabilities, and confidence scores.
- 3D Object Detection (PointPillars):
  – Pillar Feature Network: Converts sparse 3D point clouds into structured pseudo-images via voxelization.
  – Backbone: Uses 2D CNN layers to extract spatial features from pseudo-images.
  – Detection Head: Outputs 3D bounding boxes and confidence scores.

(3) C2L3-Fusion and CLOCs Framework

**Figure 2:**
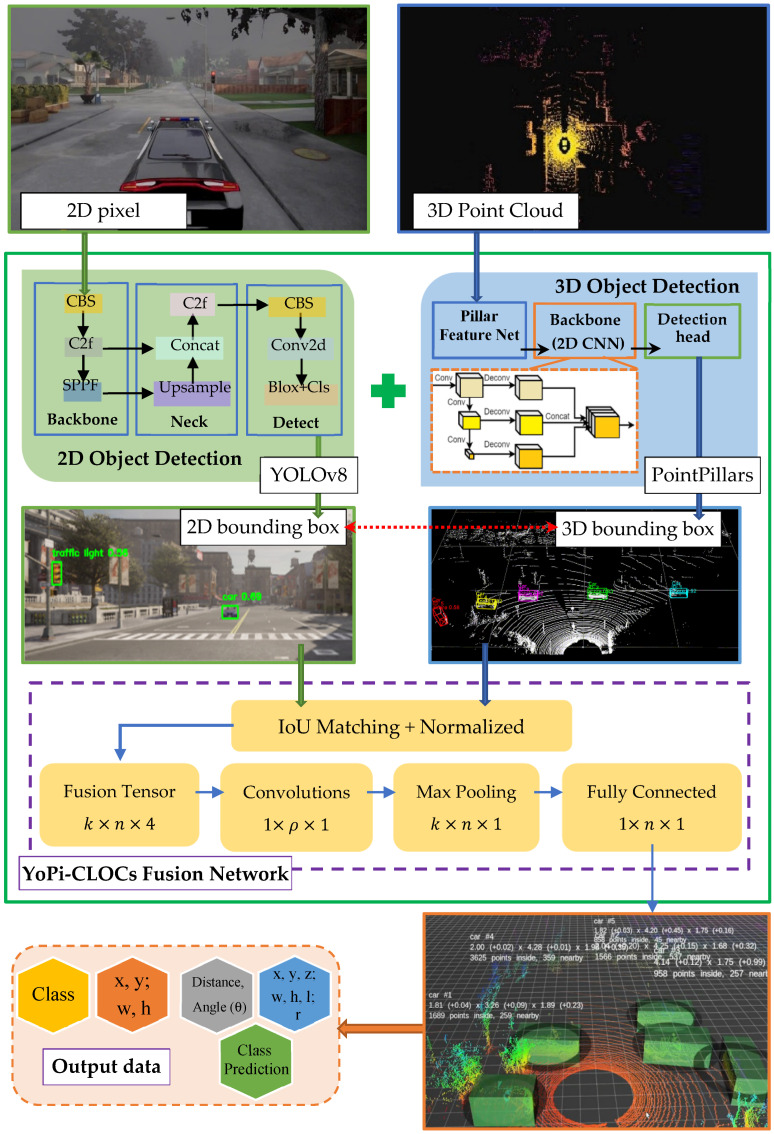
C2L3-Fusion Architecture.

The diagram in Figure 2 illustrates the structure and data processing flow of our proposed fusion model, highlighting its input, output, and integration process. In our fusion model, C2L3-Fusion (Camera-2D and LiDAR-3D Fusion), outputs from YOLOv8 and PointPillars are integrated using CLOCs (Camera-LiDAR Object Candidates), which performs the following:

- IoU Matching: Matches 2D and 3D detections based on Intersection over Union (IoU) and confidence scores.
- Fusion Tensor Processing:
  – 1 × 1 Convolutions: Reduces dimensionality while preserving critical information.
  – Max Pooling: Further reduces the feature size, generating a refined tensor.
  – Fully Connected Layers: Produces the final object class, refined 3D bounding box (x, y, z, w, h, l, θ), and confidence score.

(4) Evaluation Metrics

To assess the performance of our approach, we employ the following metrics:

- Mean Average Precision (mAP): Measures the overall detection performance.
- Intersection over Union (IoU): Evaluates spatial overlap between predictions and ground truth.
- Processing Time: Determines real-time feasibility.

(5) Experimental Setup

- Simulation Environment: The CARLA simulator is used to generate realistic urban driving scenarios.
- Training and Testing Split: The dataset is divided into 70% for training and 30% for testing to ensure robust validation.
- Hardware Implementation: Experiments are conducted on a Nvidia Jetson AGX Xavier and a high-performance GPU for comparative analysis.

### 3.2. Implementation

The implementation of the C2L3-Fusion system follows a structured approach designed to leverage multi-modal sensor data, integrating information from both 2D and 3D object detection pipelines to enhance perception for autonomous navigation. The development process consists of three major stages: data preparation, model training, and fusion-based inference.

Below is a detailed description of our C2L3-Fusion architecture, as shown in Algorithm 1:
**Algorithm 1**: C2L3-Fusion **Input**: 2D Image Data: RGB image represented as a tensor $I\in R^{H\times W\times C}$, where H, W, and C are the height, width, and color channels3D LiDAR Point Cloud: Point cloud represented as $P={\left\{\left(x,y,z,r\right)\right\}}_{i=1}^{N}$, where (x, y, z) denote spatial coordinates, r is the reflectance, and N is the number of points.**Output**:
Class Prediction:
–Object class (e.g., car, pedestrian).Bounding Boxes:
–Refined 3D bounding boxes (x, y, z, w, h, l, r).–Distance and angle (θ) or precise localization.

(1) Detection Pipelines

- 2D Object Detection: YOLOv8 is a state-of-the-art 2D object detection model optimized for speed and accuracy. It identifies object locations in the image plane and generates bounding boxes and class probabilities:
  – Backbone: Extracts low- and high-level visual features from the input image.
    (1) $$F_{2D}=Backbone(I)\;$$
  – Neck: Up-sample and combine features at different scales for robust detection.
    (2) $$F_{multi}=Neck(F_{2D})$$
  – Head (Detect): Outputs 2D bounding boxes $B_{2D}=\left\{\left(x,y,w,h\right)\right\}$, class probabilities $C_{2D}$, and confidence scores $S_{2D}$.

- 3D Object Detection (PointPillars): The PointPillars model processes raw 3D point cloud data to detect objects in the 3D space. Key components are as follows:
  – Pillar Feature Net: Converts the sparse 3D point cloud into dense pseudo-images by voxelization and feature encoding.
    (3) V=Voxelize(P)
    where $V\in R^{X\times Y\times Z}$ represents spatial voxels.
    Voxels are then into pseudo-images.
    (4) $$F_{pseudo}=FeatureNet(V)$$
  – Backbone: Applies 2D convolutional layers to extract spatial features from pseudo-images.
    (5) $$F_{3D}=Backbone(F_{pseudo})$$
  Detection Head: Outputs 3D bounding boxes $B_{3D}=\{(x,y,z,w,h,l,r)\}$ and confidence scores $S_{3D}$.

(2) CLOCs: Fusion Framework

The CLOCs framework combines 2D and 3D detection results from the previous step by aligning their features through IoU matching and score normalization:

- IoU Matching and Normalized Scores:
  – Matches 2D and 3D detections based on their IoU (Intersection over Union) and confidence scores.
    (6) $$IoU\left(B_{2D},B_{3D}\right)=\frac{Area\;of\;Intersection}{Area\;of\;Union}\;$$
  – Matched detections are weighted by their confidence scores:
    (7) $$S_{fused}=\alpha S_{2D}+\beta S_{3D},\;with\;\alpha +\beta =1\;$$
  – Produces a *Fusion Tensor* of shape k × n × 4, where k is the number of 2D detections and n is the number of 3D detections:
    (8) $$F_{fused}=Concat(F_{2D},F_{3D})$$

- Fusion Tensor Processing:
  – 1 × 1 Convolutions: Lightweight 1 × 1 convolutions and max pooling reduce dimensionality while preserving key features:
    (9) $$F_{refined}=MaxPooling(Conv1\times 1(F_{fused}))$$
  – Max Pooling: Reduces dimensionality while retaining essential features, producing a tensor of shape k × n × 1.
  Fully Connected Layer: Fully connected layers output refined bounding boxes $B_{final}$, class predictions $C_{final}$, and scores $S_{final}$.

**Figure 3:**
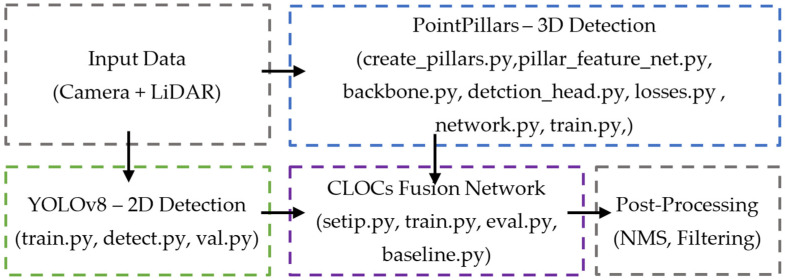
Functional block diagram of the C2L3-Fusion framework.

Figure 3 illustrates the overall architecture of the C2L3-Fusion framework, highlighting the interaction between 2D and 3D object detection components, fusion mechanisms, and post-processing techniques. This diagram provides a visual representation of the system’s modular approach to multi-modal data integration. The system processes data from two sensor sources, Camera and LiDAR, using two independent object detection models: YOLOv8 for 2D detection and PointPillars for 3D detection. A fusion neural network, CLOCs, then merges the outputs from these two models to enhance perception accuracy.

The system was validated using the KITTI dataset and further tested with real-world data collected at the University of Transport and Communications. Designed for deployment on embedded platforms such as the Nvidia Jetson AGX Xavier, the framework is optimized for real-time performance, ensuring practical applicability in autonomous vehicle systems.

(1) Data Preparation

To ensure seamless integration between 2D and 3D detection frameworks, the input data consists of synchronized 3D LiDAR point clouds and 2D RGB images. The preprocessing pipeline applies voxelization techniques to convert sparse LiDAR point clouds into structured grids, facilitating efficient feature extraction. Additionally, dataset formatting is conducted to align spatial and temporal information between different modalities, ensuring coherence in multi-sensor data fusion.

(2) Training in Component Models

The object detection process involves training two independent models, each optimized for its respective sensor modality. The 2D object detection module employs the YOLOv8 architecture, known for its real-time efficiency and high accuracy. The model is trained on a custom dataset using the Adam optimizer for 300 epochs, fine-tuning hyperparameters to maximize mean Average Precision (mAP). After training, the model undergoes rigorous validation based on Precision, Recall, and inference time before being deployed for real-time inference in static images and video streams.

For 3D object detection, the PointPillars model is utilized due to its ability to process LiDAR point clouds efficiently without requiring a traditional 3D convolutional backbone. The training pipeline consists of voxelization, features encoding using a PointNet-based network, backbone processing to extract spatial information, and a detection head that generates 3D bounding box predictions. Custom loss functions optimize Intersection over Union (IoU) and classification accuracy. The trained model is evaluated on the KITTI dataset, comparing predicted bounding boxes with ground truth annotations and computing performance metrics across 2D, BEV, and full 3D representations.

To further enhance perception, the CLOCs-based fusion network integrates detections from both modalities. This fusion framework aligns 2D bounding boxes from YOLOv8 with corresponding 3D detections from PointPillars, leveraging a multi-layer neural network to refine association strategies. The fusion model is trained on a dedicated dataset, optimizing performance through iterative backpropagation. Benchmark tests demonstrate significant improvements in detection accuracy and robustness in complex environments.

(3) Post-Processing and Optimization

**Figure 4:**
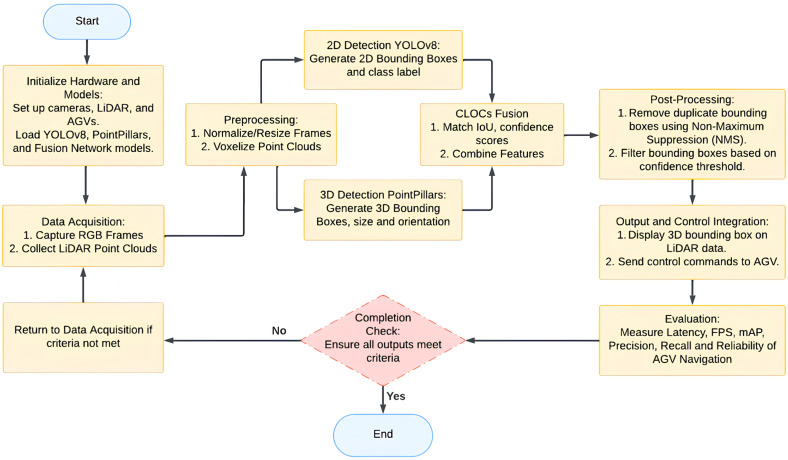
Flowchart of the real-time testing workflow.

Figure 4 presents the real-time testing workflow for the C2L3-Fusion model, demonstrating the sequential processing stages from sensor data acquisition to final output generation. The diagram showcases the efficiency of the proposed method in handling real-world conditions, optimizing computational performance, and ensuring robust object detection for autonomous navigation.

Following detection and fusion, a post-processing stage refines object predictions and eliminates inconsistencies. Non-Maximum Suppression (NMS) filters redundant bounding boxes, ensuring that only the most confident detections are retained. Additional filtering techniques enhance localization accuracy, addressing occlusions and reducing false positives. The final output comprises a robust multi-modal detection pipeline capable of real-time operation, making it suitable for deployment in autonomous vehicle applications.

By adopting a systematic and data-driven approach, the C2L3-Fusion framework effectively integrates multiple perception modalities, demonstrating superior performance in occluded, sparsely populated, and highly dynamic environments. Through the synergy of YOLOv8, PointPillars, and CLOCs, this fusion-based detection pipeline significantly enhances the vehicle’s ability to perceive and navigate its surroundings with high precision and reliability.

## 4. Results and Discussion

### 4.1. KITTI Dataset Results

**Figure 5:**
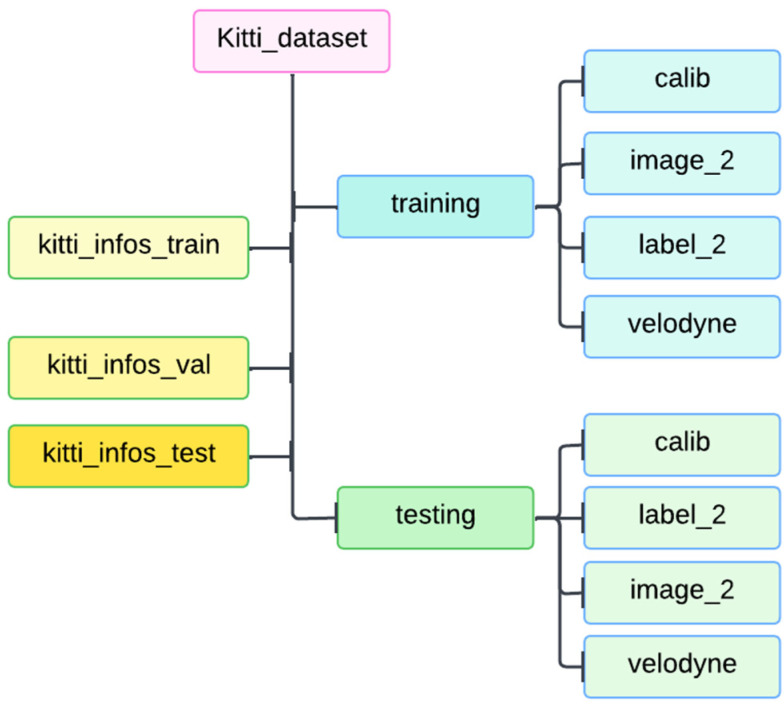
Structure of the KITTI dataset, showing the organization of training and testing data, including RGB images, point clouds, and labels.

The C2L3-Fusion model was evaluated using the KITTI dataset [25], which is a standard benchmark for autonomous driving tasks. The dataset structure is depicted in Figure 5, comprising RGB images, LiDAR point clouds, and labels for ground-truth annotations, used for both training and testing purposes.

Some of the best detection methods now have been selected to make comparisons. In Table 1, all the models are evaluated on the KITTI 3D object detection benchmark [25]. The KITTI dataset consists of 7481 training samples and 7518 testing samples in the object detection task. The training data are divided into a training set with 3712 samples and a validation set with 3769 samples. The results on the validation set and test set are evaluated with the average precision calculated by 40 recall positions. All the models are evaluated in three settings: easy, moderate, and hard.

**Figure 6:**
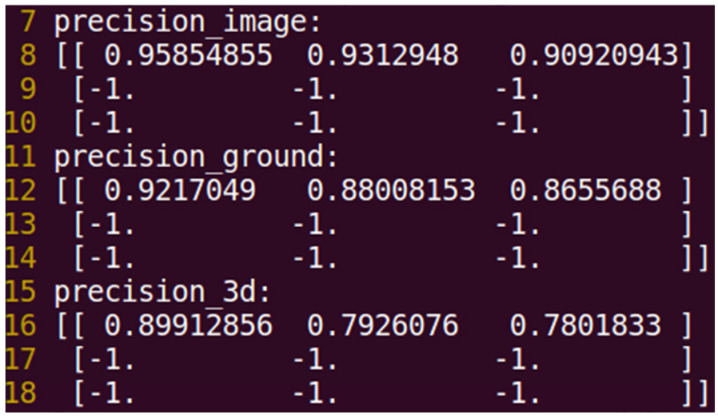
The developed model evaluation results on easy, moderate, and hard setting after 15 epochs of training.

After training for 15 epochs, the C2L3-Fusion model demonstrated significant improvements compared to the baseline PointPillars approach. The evaluation considered three difficulty levels—easy, moderate, and hard—based on object occlusion and distance. The developed model’s performance in terms of Average Precision (AP) is illustrated in Figure 6.

Table 1 presents a comparative analysis of our proposed method against baseline 3D object detection approaches, including PointPillars and CLOCs, as well as other state-of-the-art methods under the same evaluation conditions. The evaluation was conducted on the KITTI dataset, focusing on the car category with an IoU threshold of 0.7. The results demonstrate that our C2L3-Fusion model achieves superior object detection accuracy and more reliable 3D bounding box localization.

### 4.2. Real-World Testing Results

Following the successful evaluation of the KITTI dataset, the proposed fusion model was further validated using real-world data collected in a controlled environment. The dataset was recorded using the Ouster OS1-64 LiDAR sensor, managed via the rosbag library, ensuring high-fidelity data synchronization for accurate perception and decision-making in autonomous navigation.

The fusion model was deployed on an Nvidia Jetson AGX Xavier embedded computing platform, featuring a 512-core CUDA GPU, 64 Tensor cores, and 64 GB of RAM. This setup provides the computational power required for real-time inference, enabling low-latency multi-modal sensor data processing.

To integrate the model into an autonomous navigation system, the Robot Operating System (ROS) was utilized as the middleware framework. ROS facilitates communication between perception, planning, and control modules in real-time, ensuring system modularity and scalability.

**Figure 7:**
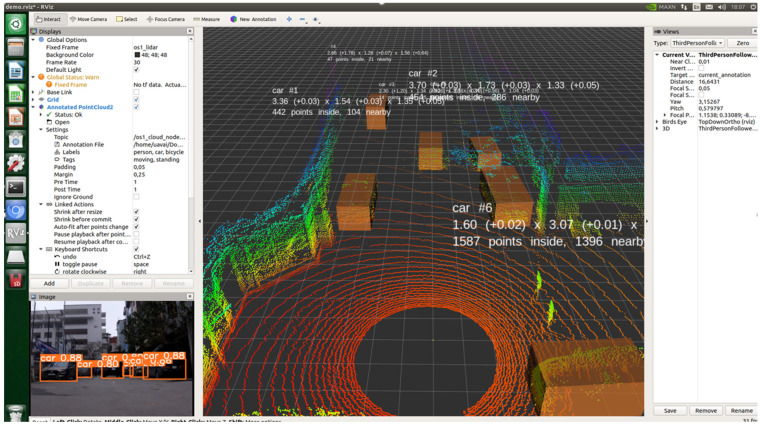
Real-time visualization of object detection using custom-collected data using rosbag and a LiDAR-based sensor setup (note that commas in the left and right panels denote decimal places).

The visualization results in Figure 7 illustrate the model’s ability to generate 3D bounding boxes around detected objects in real-world scenarios. The detection framework effectively identifies multiple vehicles and structures, with only minor observations noted for further enhancement:

- Bounding box precision: The 3D localization is generally accurate, though slight alignment variations may occur in cases involving distant or partially occluded objects.
- Classification confidence: In rare instances, especially near image boundaries, detected objects may exhibit minor uncertainties in classification or bounding box definition.
- Benchmark dataset differences: Although the model shows reliable performance in controlled scenarios, natural variations in real-world sensor data quality and dynamic factors (such as moving pedestrians or vehicles) pose additional considerations compared to benchmark datasets like KITTI.

Overall, the fusion model maintains a real-time processing rate of 30+ FPS, demonstrating strong applicability for autonomous perception tasks. Future refinements, such as enhanced LiDAR–camera calibration or optimized post-processing, could further improve detection accuracy and robustness in complex urban environments.15

## 5. Conclusions and Discussion

This paper presented C2L3-Fusion, a novel sensor fusion approach that integrates LiDAR and camera data to enhance object detection and 3D bounding box localization for autonomous systems. By leveraging complementary sensor modalities, our model effectively addresses limitations inherent in single-sensor approaches, improving robustness in diverse environmental conditions.

The experimental evaluation demonstrated that C2L3-Fusion outperforms existing LiDAR-only and LiDAR + RGB fusion methods, achieving higher accuracy in detecting and localizing objects. Real-world testing further validated the model’s effectiveness, with the system maintaining stable performance across varying lighting conditions, occlusions, and dynamic environments. Additionally, the model’s ability to operate at over 30 FPS ensures its suitability for real-time autonomous applications.

However, challenges remain. While fusion improves detection accuracy, computational complexity increases, potentially limiting deployment on lower-power embedded systems. Additionally, the reliance on high-quality sensor calibration and synchronization is critical, as misalignment between LiDAR and camera data can degrade performance. Addressing these issues requires optimization strategies, such as lightweight neural architectures and advanced calibration techniques.

Future work will focus on (i) evaluating the computational efficiency of C2L3-Fusion across common embedded platforms, (ii) improving real-time efficiency of the fusion network using other models (e.g., transformer-based architectures), (iii) enhancing generalization to different weather conditions (e.g., rain, fog, or snow) or operating environments (day versus night, or urban versus highway scenarios), and integrating additional sensor modalities (e.g., radar) to further strengthen robustness of the system. The findings of these will contribute to the advancement of autonomous perception, providing a reliable framework for real-world deployment in self-driving applications.

## Figures and Tables

**Table 1:** Detection performance of Car category on the KITTI validation set with AP (3D) with IoU = 0.7.

Method	Input Data	Easy	Moderate	Hard
MonoDTR [16]	Camera	21.99	15.39	12.73
PointPillars [17]	LiDAR	79.05	74.99	68.30
AVOD [20]	LiDAR + RGB	81.94	71.88	66.38
CLOCs_PVCas [14]	LiDAR + RGB	88.94	80.67	77.15
C2L3-Fusion (ours)	LiDAR + RGB	**89.91**	**79.26**	**78.01**

## Data Availability

Datasets generated during the study are available from the corresponding authors on reasonable request.
